# Dynamics Correlation Network for Allosteric Switching of PreQ_1_ Riboswitch

**DOI:** 10.1038/srep31005

**Published:** 2016-08-03

**Authors:** Wei Wang, Cheng Jiang, Jinmai Zhang, Wei Ye, Ray Luo, Hai-Feng Chen

**Affiliations:** 1 State Key Laboratory of Microbial metabolism, Department of Bioinformatics and Biostatistics, College of Life Sciences and Biotechnology, Shanghai Jiaotong University, 800 Dongchuan Road, Shanghai, 200240, China; 2 Departments of Molecular Biology and Biochemistry, Chemical Engineering and Materials Science, Biomedical Engineering, University of California, Irvine, California 92697-3900, USA; 3 Shanghai Center for Bioinformation Technology, 1278 Keyuan Road, Shanghai, 200235, China

## Abstract

Riboswitches are a class of metabolism control elements mostly found in bacteria. Due to their fundamental importance in bacteria gene regulation, riboswitches have been proposed as antibacterial drug targets. Prequeuosine (preQ_1_) is the last free precursor in the biosynthetic pathway of queuosine that is crucial for translation efficiency and fidelity. However, the regulation mechanism for the preQ_1_ riboswitch remains unclear. Here we constructed fluctuation correlation network based on all-atom molecular dynamics simulations to reveal the regulation mechanism. The results suggest that the correlation network in the bound riboswitch is distinctly different from that in the apo riboswitch. The community network indicates that the information freely transfers from the binding site of preQ_1_ to the expression platform of the P3 helix in the bound riboswitch and the P3 helix is a bottleneck in the apo riboswitch. Thus, a hypothesis of “preQ_1_-binding induced allosteric switching” is proposed to link riboswitch and translation regulation. The community networks of mutants support this hypothesis. Finally, a possible allosteric pathway of A50-A51-A52-U10-A11-G12-G56 was also identified based on the shortest path algorithm and confirmed by mutations and network perturbation. The novel fluctuation network analysis method can be used as a general strategy in studies of riboswitch structure-function relationship.

Riboswitches, first discovered ten years ago^1^,^2^,^3^, are a class of genetic regulatory elements found in numerous evolutionarily distant bacteria, with counterparts in plants, fungi, and archaea^4^. These intracellular sensors of metabolite always locate at the 5′ end of mRNA and function as allosteric regulators to exert control of transcription, translation, splicing, and RNA stability. More than 20 types of riboswitches^5^,^6^,^7^ have been found based on bioinformatics prediction and experiment verification^8^. A riboswitch can often be conceptually split into two parts, the aptamer domain and the expression platform. The aptamer domain, which contains the specific evolutionarily conserved ligand-binding sequences, senses the metabolites with high selectivity and affinity. The expression platform enables regulation of the downstream coding sequences^9^,^10^. When the metabolite concentration exceeds a threshold level, these riboswitches start their function of genetic switch upon binding with the ligands that induce conformation changes. The expression platform locates at the downstream of the aptamer, serving as a switch and conducting different regulatory strategies. The most common modulation strategy is to form a transcription terminator by sequestering the Shine-Dalgarno (SD) sequence to prevent binding of the ribosome. Due to their fundamental importance in bacteria gene regulation, riboswitches have been proposed as antibacterial drug targets^11^.

The preQ_1_ riboswitch is a member of a large subset of riboswitches that selectively recognize purine and its derivatives^12^,^13^. It is involved in the regulation of queuosine (Q) biosynthesis and transport. The preQ_1_ is the last free precursor in the biosynthetic pathway of prokaryote organism before insertion into the tRNA wobble position^14^. The NMR solution structure of preQ1 II riboswitch was released in 2014 (pdb code: 2MIY)^15^, which reveals the key functionality of the embedded hairpin for riboswitch in recognition of preQ1. The structure (Fig. 1) contains four helices and two junctions: helix P1 with G1-G7 and C20-C27, helix P2 with C13-U19 and A50-G56, helix P3 with G37-G40 and C45-C48, junction J2-3 from C8 to G12, junction J2-4 from A27 to C36, and helix P4 from G57 to G68. Helix P4 is approximately perpendicular to the coaxially stacked P2 and P3 helical axes, and is highly flexible for ligand binding. Two flanking adenines of helix P4 play crucial roles in locking the ligand and sequestering the ribosome binding site. PreQ_1_ binds at the three-way junction where helices P2, P3 and P4 interchange.

Additional functional study was also available in the literature to examine the roles of P2, P3, and P4 helices, by focusing on multiple disruptive and compensatory mutations in these secondary structures (listed in Table 1)^16^. The disruptive mutation in P2 (M1) decreases the ligand binding affinity while the compensatory mutation (M2) partially restores the ligand binding affinity. This suggests that the sequence of P2 might not conserve. Disruptive mutation M3 and compensatory mutation (M4) are located in P3. M3 abolishes the preQ1 binding but M4 does not restore the preQ1 binding. This indicates that the P3 nucleotides mutated are strictly conserved due to the presence of SD and anti-SD sequences, and this region modulates the ligand binding. Finally the disruptive mutant in P4 (M5) abolishes the preQ1 binding and the compensatory mutation in P4 (M6) fully restores the ligand binding. This suggests that the sequence of P4 might not conserve even if the secondary structure of P4 is conserved.

Based on these structural and functional evidences, a new mechanism was proposed to understand how the preQ1-II riboswitch exquisitely controls the queuosine metabolism^15^. These experimental data lay down the foundation for deciphering the molecular mechanisms of preQ1 riboswitch, and also prompt many further questions, for example, (1) how does the information transfer from preQ1 to the expression platform? (2) what is the molecular mechanism of switch regulation? and (3) what are the regulation pathways in riboswitch? To answer these questions, we studied the dynamics features of the wild type and mutant riboswitches with all-atom molecular dynamics (MD) simulations. Based on the ensemble of trajectories, we constructed nucleotide fluctuation correlation networks, which was previously used to illustrate the allosteric phenomenon of aminoacyl-tRNA synthetase^17^. Given the analyses and comparisons of the networks between apo and bound riboswitches, a binding induced switch pathway was proposed for transferring the regulation information from the binding site to the expression platform.

## Materials and Methods

### Molecular dynamics simulation

The atomic coordinates of the preQ1-riboswitch complex were extracted from RCSB (PDB code: 2MIY)^15^. M1, M3, and M5 are disruptive mutants of the P2, P3, and P4 helices, respectively. M2, M4, and M6 are complementary mutants for M1, M3, and M5, respectively^12^. MS1(A35G) and MS2(A50G) are the mutants mentioned in the previous study to reveal the screw cap function of the P4 helix^15^. These mutant structures were constructed using Sybyl-X^18^. All structural visualizations were conducted in PyMOL 1.7^19^.

All initial structures were first minimized in SYBYL^®^-X 2.1.1 to eliminate any possible overlaps or clashes. AMBER12 was used to perform efficient simulations with periodic boundary conditions^20^. Hydrogen atoms were added using the LEaP module of AMBER12. Counter-ions were used to maintain system neutrality. All systems were solvated in a truncated octahedron box of TIP3P waters with a buffer of 10 Å. The pairwise interactions (van der Waals and direct Coulomb) were computed with a cutoff distance of 8 Å. Particle Mesh Ewald (PME) was employed to treat long-range electrostatic interactions in AMBER12^21^. The ff99SB force field was used for the intramolecular interactions. The SHAKE algorithm^22^ was used to constrain bonds involving hydrogen atoms. All MD simulations were accelerated with the CUDA version of PMEMD in GPU cores of NVIDIA^®^ Tesla K20. Up to 20000-step steepest descent minimization was performed to relieve any structural clash in the solvated systems. This was followed by a 400-ps’ heating up and a 200-ps’ equilibration in the NVT ensemble at 298 K. The Langevin thermostat was used in the preparation runs with a friction constant of 1 ps^−1^ and the Berendson thermostat with default setting was used in the production runs^19^,^23^.

To compare the difference between preQ_1_ bound and free riboswitches, three independent trajectories of 100 ns each were simulated. 1.8 μs trajectories in all were collected for the wild type and mutant systems at 298 K, taking about 1200 GPU hours. Detailed simulation conditions are listed in Table 1.

### Data analysis

Tertiary contact assignment was handled with in-house software^24^,^25^. The nucleotides and preQ1 are in hydrophobic contact when mass centers of their bases and preQ1 are closer than 6.5 Å for the complex. A previous study has shown that charge-to-charge interactions up to 11 Å were found to contribute to RNA/protein binding free energies^26^. Thus, electrostatic (i.e. charge-charge) interactions are assigned when the distance (centers of mass) between the base of preQ1 and the phosphate for riboswitch is less than 11 Å. Hydrogen bond is defined that the distance between two polar heavy atoms either with a hydrogen atom is less than 3.5 Å.

The energy landscape was mapped by calculating normalized probability from a histogram analysis, and plotted with Origin 8.5, which was also employed to plot the diagrams in this paper. For each simulation, sampling was conducted every 10 ps (10000 snapshots for 100 ns’ simulations). Radius of gyration (Rg) and root mean standard deviation (RMSD) were both separated into 8 bins. The energy landscape was plotted among these 64 (8 × 8) bins. Average structures were extracted from the structure ensembles of the lowest energy^26^,^27^,^28^,^29^.

### MM/PBSA energy calculation

Free-energy calculation together with MD simulations can provide qualitative predictions of riboswitch–preQ1 binding energies. The free energy of binding (Δ*G*_*bind*_) is estimated by


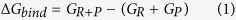


where *G*_*R + P*_, *G*_*R*_, and *G*_*P*_ is the free energies of the complex, the isolated riboswitch and preQ1, respectively. In the MM/PBSA approach^27^,^30^,^31^,^32^, each free energy term in Eq. 1 is calculated as


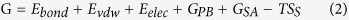


where *E*_*bond*_ is the molecular mechanics bond energy, which is the sum of bond, angle and dihedral energies; *E*_*vdw*_ is the molecular mechanics van der Waals energy contribution; *E*_*elec*_ is the molecular mechanics electrostatic energy; *G*_*PB*_ and *G*_*SA*_ are polar and non-polar contributions to the solvation energy; T is the absolute temperature and *S*_*S*_ is the solute entropy. All the binding free energies were calculated using the PB model in MMPBSA^27^,^30^,^31^,^32^. Considering that the equilibration period may affect the result, only the last 50 ns of MD trajectories was used to calculate the binding free energy taking snapshots every 50 ps.

### Correlation and Network Analyses

Every nucleotide was divided into two nodes (backbone and base) for more detailed analysis of their fluctuation dynamics. The fluctuation correlation between any pair of nodes was calculated as


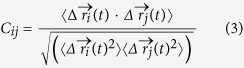


where 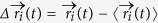, 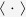 represents a time averaging, and 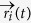 is the position of node *i* at time *t*. These elements were conveniently organized as a covariance matrix for a simulated system. In the current study, the covariance matrix for each system was constructed using snapshots (every 2 ps) in the last 50 ns of all simulated trajectories. Besides nodes, “edge” that transfers allostery information from one node to another is also a key concept in network construction. An edge is defined between any two nodes without covalent bond but with heavy atoms closer than 4.5 Å over 75% sampling time. The strength of the edge is defined as the absolute value of the inter-node correlation (*C*_*ij*_) between node *i* and *j*. The number of connected edges at each node is defined as the degree of the node. Correlation-weighted degree, which is the summation of strengths of all edges connected to a given node, indicates the importance of the node. After the network construction, network topological analyses were performed using Cytoscape3.1.1^33^. The Floyd-Warshall algorithm was used to calculate the shortest path between any two nodes in the network. For community analyses, the Girvan-Newman algorithm was utilized. Most of the network analysis tools utilized here were developed by the Luthey-Schulten Group^17^,^34^,^35^.

### Characteristic Path Length (CPL) Analysis

The CPL is defined as the average distance between all pairs of nodes in the network. Distance is the length of shortest path between two nodes. The CPL was computed as


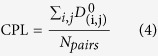


where 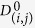 is the shortest distance between nodes i and j; *N*_pairs_ is the number of all pairs of nodes connected by a path in the system. The contribution of a node *k* to communication within the network is found by recalculating the CPL after removing node *k* (*CPL*_*k,*rem_) from the network. The difference in CPL (Δ*CPL*_*k,*rem_ = *CPL*_*k,*rem_ − *CPL*) was used to identify hub nucleotides in switch regulation, which has large CPL values. Hub nucleotides play key roles in switch regulation. This method has been used previously to predict residues important in allosteric signaling within protein families^36^.

## Results and Discussion

Analysis of structural evolutions with respect to the initial structures shows that 100 ns simulations are sufficient for the equilibration of wild type and mutant systems at 298 K (supplementary Figure S1). Root mean squared fluctuations of C5′, C1′, N3, N1 for apo and bound riboswitches are shown in supplementary Figures S2–S5, respectively. In general, the variations of these atoms for the bound state are lower than those of the apo state, especially for the nucleotides around the binding pocket. Two peaks between A30 and G47 are 2 Å higher than other parts in the apo state, suggesting that J2-4 and P4 are much more flexible in the apo state than in the bound state. The pseudoknot region in the apo riboswitch also shows higher fluctuation than that in the bound riboswitch. This indicates that the expression platform becomes slightly unstable upon dissociation of preQ_1_.

To confirm the reliability and robustness of MD simulations, the binding free energies between preQ1 and riboswitch for the wild type and mutants are calculated with MMPBSA and shown in supplementary Table S1^27^,^30^,^31^,^32^. The binding free energy of the G5C/U6A mutant is about 5 kcal/mol higher than that of the wild type. This result is in accord with the experiment that the G5C/U6A mutant introduces a 100-fold decrease in binding affinity. For the reversed mutant of M2, the binding free energy is almost same to that of the wild type. For the disruptive mutants of M3 and M4, the binding free energies are about 9 kcal/mol higher than that of the wild type. The correlation coefficient is 0.869 between measured pK_D_ and binding free energies and shown in supplementary Figure S6. In summary, the MD simulation and binding free energy analysis are in good agreement with previous experiments^15^.

**Figure 2:**
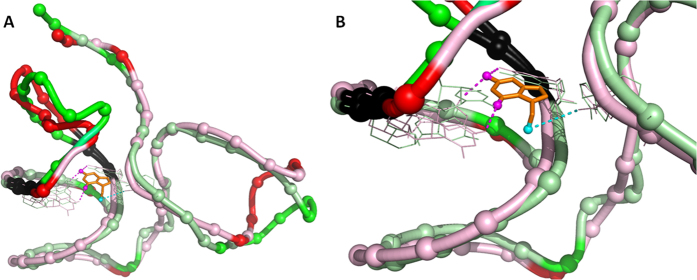
Identification of driver and anchor atoms with apo and bound riboswitches. (**A**) Global view. (**B**) Zoomed-in at the preQ1-binding region. The driver atom is labeled as a cyan sphere and the anchor atom is labeled as a magenta sphere. The base that interacts with the driver atoms is A50. Bases that interact with anchor atoms are G7 and U19. Riboswitch is represented as ribbon, with the backbone phosphate atom embedded as spheres. The ligand is depicted as stick. The dark color of the ribbons indicates regions with large conformational changes, where the global backbone displacement (GBD) of corresponding pairs is greater than a preset distance (3.5 Å). Side chains depicted as lines are shown only for residues in contact with the riboswitch’s own ligand; the residues are highlighted in dark ribbon if the local structural environment (LSE) change is below a threshold value (1.0 Å). Identified hydrogen bonds are highlighted with a dashed tube (yellow) between a host base atom (tube) and a ligand atom (sphere). The driver atom and anchor atom were identified with given operational structural criteria with a GBD of 3.5 Å and LSE of 1.0 Å.

Kang *et al*. reports that preQ1 binds at the three-way junction where P2, P3 and P4 helices interchange and sequester the ribosome binding site^37^. Furthermore, the ribosome binding site of P3 is about 30 Å away from the preQ1 binding site. The binding of preQ1 thus causes a long-rang conformation change and plays a key role in the downregulation of gene expression. Therefore, an allosteric regulation mechanism may be used to explain the preQ1 riboswitching function. Recent work publishes a method to identify allosteric site^38^. Furthermore, Nussinov *et al*. reports that driver and anchor atoms exhibit specific interactions with the host protein, with the driver atoms mainly responsible for the allosteric efficacy and the anchor atoms for the binding affinity^39^. In order to identify the possible driver and anchor atoms for preQ1 riboswitch, the reported methods^39^ were used to assign the possible driver atoms and the results are shown in Fig. 2. These functional atoms might play key roles in the allosteric switching function by the riboswitch.

To further illustrate the conformational difference, the landscapes of distance differences between apo and bound states for the riboswitch were analyzed and shown in supplementary Figure S7. The landscapes may reflect the relative conformational changes of the riboswitch backbone. The dark blue areas show that the differences between J2-4 and P4, P3 are negative. This indicates that the P3, P4 and J2-4 regions are stretched upon preQ1 dissociation. The other regions of riboswitch are not changed dramatically based on the landscape analysis.

### Correlation Networks of Apo and Bound Riboswitches are Different

Structural analysis suggests that allosteric regulation may exist in the preQ1 riboswitch. In order to reveal the molecular mechanisms of the regulation, a network analysis was used to analyze the nucleotide fluctuation correlation in this study. To construct the correlation network, the covariance matrices were first calculated for the riboswitch. The differences of correlation between apo and bound riboswitches (C_apo_ − C_bound_) are shown in supplementary Figure S8, which indicates that the correlations between P4 and J2-4 are much lower in the bound state than those in the apo state. These regions are more compact in the bound state. On the contrary, the regions of J2-3 and P3 are highly correlated in the bound state. This demonstrates that the expression-platform regions are strongly correlated with preQ1.

**Figure 3:**
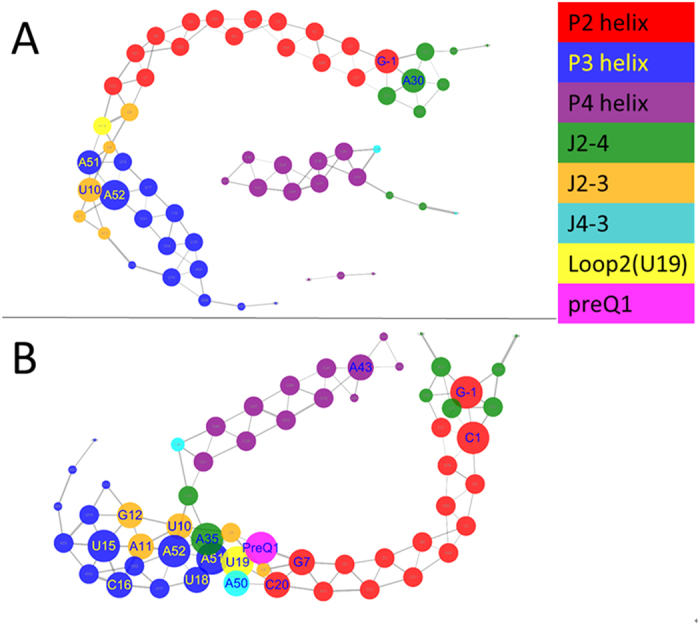
Correlation networks of apo and bound riboswitches. (**A**) Apo-riboswitch. One node represents a nucleotide. The strength of the edge is defined as the absolute value of the inter-node correlation between two nodes. Different colors represent different secondary domains. Nodes with degree larger than four have been marked with its nucleotide names. The size of node represents the value of degree. (**B**) Bound riboswitch.

Based on the covariance matrices, fluctuation correlation networks were constructed. The topology parameters of bound and apo networks are listed in Table 2. The network centralization and density of the bound state are higher than those of the apo state. The average numbers of neighbors and heterogeneity for the bound network are also higher than those of the apo network. The correlation networks for bound and apo states are shown in Fig. 3. It shows that the number of nodes with degree higher than four (more than four edges) is 17 in the bound network and more than that of the apo network (5). The important hub nodes that play key roles in the switch, such as A50, A51, U19, A35, A52, U10, and A11, have higher degrees in the bound network. Most of them directly interact with preQ1. Interestingly, the degrees of U19, A35, and A50 significantly decrease upon the dissociation from preQ1. Furthermore, the apo network splits into three pieces. In order to evaluate the robustness of the above analysis, the correlation networks of three independent trajectories for both apo and bound riboswitches were each analyzed and are shown in supplementary Figure S9. The figure suggests that the apo networks from different trajectories are similar, but they are significantly different from the three bound networks. A Kolmogorov–Smirnov (KS) *p-*test was further used to statistically compare the networks from apo and bound riboswitches, with correlation-weighted degree as input data (supplementary Table S2). All trajectories from the same state yield high *p*-values (*P* > 0.11), indicating similarity in the correlation-weighted degree’s distributions. However the *p*-values of all trajectories between bound and apo states are very low (*P < 0.035*), indicating significant difference. These analyses show that this approach constructing dynamics correlation network from molecular dynamics simulation is robust and reliable.

### Characteristic Path Length Analysis

**Figure 4:**
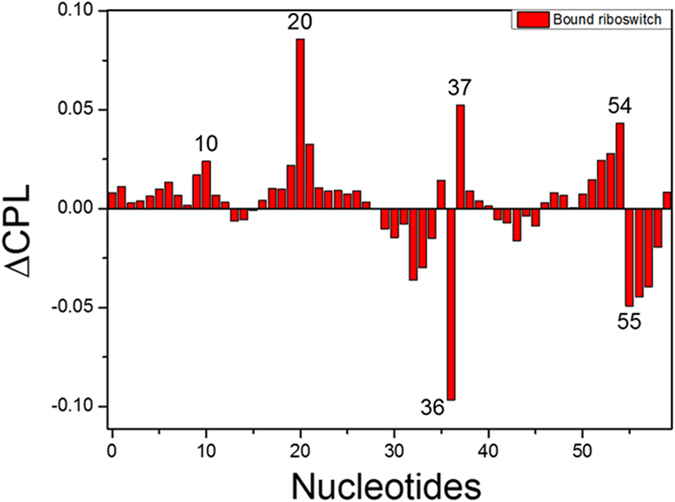
Differences in characteristic path length (CPL): the change in CPL upon edge removal for each nucleotide in the network of the bound riboswitch. The nucleotides with significant increase or decrease in CPL are labeled.

To identify nucleotides that have the largest effect on information communication in the networks, the change in characteristic path length is calculated upon removing all contacts from a given nucleotide to any nucleotide in this network and keeping all other contacts in the network intact. Nucleotides that significantly increase or decrease the edge in characteristic path length are labeled in Fig. 4, which shows that two nodes (U10 and C20) are nearby the binding pocket. These nucleotides may facilitate the binding information transfer to the expression platform. C36 and G37, which are close to A35, also play key roles in maintaining the integrity of the network. In addition, removal of nucleotides in the P3 helix shows higher characteristic path length. These suggest that P3 helix plays a key role in the regulation.

### Different Networks Can Be Split into Different Communities

**Figure 5:**
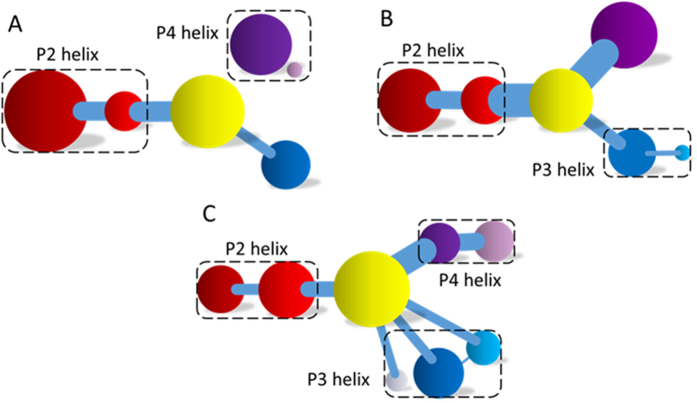
Community networks for apo and bound riboswitches. (**A**) Apo riboswitch. Red represents the P2 helix, yellow the preQ1-binding pocket, blue the P3 helix, and purple the P4 helix. (**B**) Bound riboswitch. (**C**) Modified bound riboswitch where all edges between preQ1 and the riboswitch are deleted.

The analysis of correlation network shows that the networks are significantly different between apo and bound riboswitches. To reveal the information transfer pathway, the Girvan-Newman algorithm was used to split the network into communities. The community network is shown in Fig. 5A,B, which shows that the bound network is clustered into one single community. The information flow can freely transfer from the binding site of preQ1 to the expression platform of P3. However, the community network is significantly changed and P4 is isolated from the main community network upon dissociation from preQ_1_. Information flow can hardly transfer from the binding site to the expression platform. These results are in agreement with those of the correlation network analysis (shown in Fig. 3). Note too that the information intensity of the bound community network is also stronger than that of the apo community network, especially for the community of the binding pocket. Moreover, structural analysis indicates that there are three hydrogen bonds, four hydrophobic interactions, and nine electrostatic interactions between preQ1 and riboswitch, with population higher than 40% (supplementary Figure S10). These indicate fairly strong binding interactions. Therefore a hypothesis of “binding induced allosteric switching” can be used to explain the regulation mode for preQ1 riboswitch.

### Modifications Used to Perturb the Community Network

In order to validate this hypothesis, modification to weaken the interactions between preQ1 and riboswitch was used to study the effects on the community network. Without carrying out another MD simulation, this modification was realized by deleting the edges between preQ1 and riboswitch nodes in the network. Interestingly the modification leads to significant repartition among the community network as shown in Fig. 5C. This figure shows that P3 is divided into three distinct communities with little information transfer among them. Thus the efficiency of information transfer is decreased. This finding confirms that the interactions between preQ1 and riboswitch indeed influence the community network and further support our hypothesis of binding induced allosteric switching.

### Mutant Validation of Binding Induced Allosteric Switching

**Figure 6:**
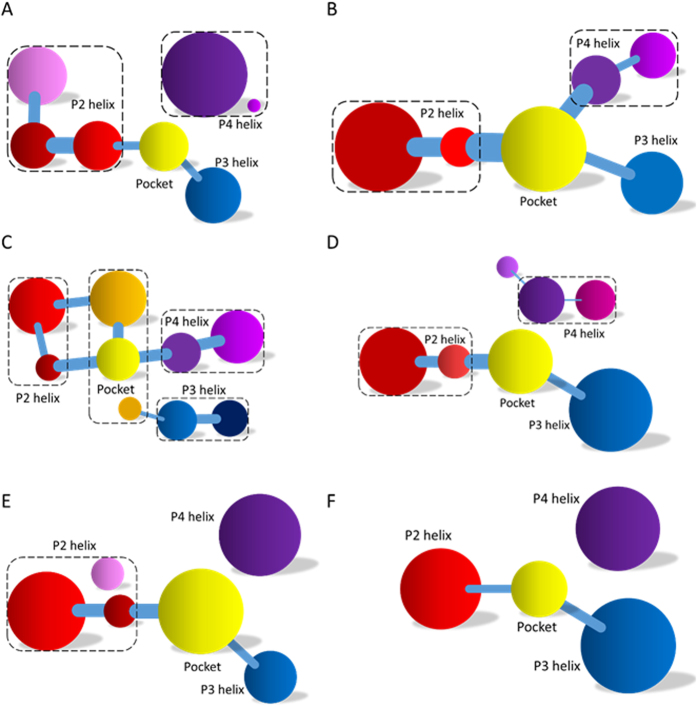
Community networks of all tested mutants. (**A**) M1; (**B**) M2; (**C**) M3; (**D**) M4; (**E**) A50G; and (**F**) A35G.

To furthermore evaluate the hypothesis “binding induced allosteric switching”, the networks of these mutants were constructed and compared with that of the wild type. The networks of the M1 and M2 mutants are shown in supplementary Figure S11, which indicates that the network of M1 is separated into two subsets. One is the main part composed of P2 and P3 helices, the rest is composed of the P4 helix. In addition, the connection strengths between preQ1 and riboswitch are reduced. That is, without the wild type P2 helix, preQ_1_ cannot function well in regulating RBS. Figure S9 also indicates that the network of the compensatory M2 mutant is well connected and U10, U19, A35, and A51 are hub nodes. This shows that the sequence of P2 might not conserve. Comparison with the wild type network shows that the network of M1 is very similar to that of the apo riboswitch and the network of M2 is similar to that of the bound riboswitch. Structural analysis shows that the interactions between preQ1 and riboswitch in the M1 mutant are significantly weaker than those in the wild type (supplementary Figure S12), while these interactions in the M2 mutant are similar to those in the wild type. This further confirms that M1 destroys the information transfer while M2 restores the information transfer. The communities of M1 and M2 are shown in Fig. 6A,B. These analyses show that the information could hardly transfer from the binding pocket of preQ1 to the expression platform of P3 in the M1 mutant. However, the information can freely transfer in the M2 mutant.

M3 and M4 mutations on P3 were analyzed similarly. Their networks are shown in supplementary Figure S13. Surprisingly, the network for the M3 mutant is completely different from that of the wild type whether it is in the apo or the bound states. The hub nodes of U10 and A50 disappear. The changes in the network topologies and hub nodes interrupt the information transfer pathway. Network topology is also changed upon the M4 mutation. The hub nodes of U10, U19, and A35 identified in the wild type network are completely gone. In order to compare the information transfer pathway with the wild type network, the communities of M3 and M4 mutants were analyzed (Fig. 6C,D). These figures indicate that bottlenecks for information transfer exist in the network. In summary, M3 and M4 mutants confirm that the weakened binding interaction destroys the switch regulation pathway in the riboswitch. Furthermore, structural analysis indicates that the interactions between preQ1 and riboswitch for M3 and M4 mutants are much weaker than those for wild type (supplementary Figure S12). Finally the network and community analyses for M5 and M6 mutants are shown in supplementary Figures S14–S15. Similar conclusions can be drawn found for the both mutants. These results further verify the hypothesis of binding induced allosteric switching for preQ1 riboswitch.

### PreQ1-Binding Induced Allosteric Switch Regulation Pathway

**Figure 7:**
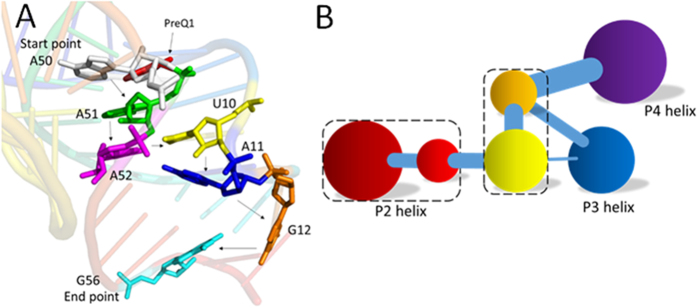
(**A**) Shortest pathway from the binding pocket to the expression platform. (**B**) Community network after removing all connections of A11.

The network and community analyses of the wild type and mutants confirm that the preQ1 binding induces its switching function. Next, it is natural to identify the allosteric pathway based on the network analysis. Shortest path algorithm was used to identify the allosteric pathway between the binding pocket and the expression platform^40^. The pathway in the preQ1 bound riboswitch is A50-A51-A52-U10-A11-G12-G56 (shown in Fig. 7A). However, we could not find a similar pathway in the apo riboswitch. This indicates that A50, A51, A52, U10, A11, G12, and G56 are key nodes for information transfer in the preQ1 riboswitch. In order to confirm their critical roles on the allosteric regulation, A50 was mutated to study its effect. The network of A50G mutant is shown in supplementary Figure S16. This figure shows that the network is split into two parts and P4 is isolated from the rest. This indicates that A50 is one of the hub nodes for this network. The community network of mutant A50G is shown in Fig. 6E, which also shows that the information flow for A50G cannot freely transfer from the binding pocket to the expression platform. This further confirms the importance of A50 on the allosteric regulation and is consistent with the experimental observation that A50G results in a 280-fold decrease in binding affinity for preQ1^37^. Next we analyzed mutant A35G that disrupts the hydrogen bond with A51^37^. The network analysis shows that A35G significantly changes the community network and perturbs the transfer of information (shown in Fig. 6F). This is also in accord with experiment that A35G introduces a 9-fold decreases in binding affinity for preQ1^37^.

In order to further verify the allosteric pathway, network perturbation was used to study the effect of A11, which is the linkage node between the binding pocket region and P3, the bottleneck of information transfer. Without carrying out another MD simulation, the perturbation was realized by weakening the edges between A11 and any nucleotide in the network. The community network is shown in Fig. 7B. The community is significantly repartitioned that the binding pocket regroups into two communities and has several nodes shared with P3. This change decreases the effectiveness of information transfer, confirming that A11 plays a key role in this allosteric switch.

### Comparison with Previous Experiments

The NMR structure shows that U19 and C8 can form stable hydrogen bonds with preQ1^15^. Three stable hydrogen bonds for U19(O4)/preQ1, C8(N3)/preQ1, and C8(O2)/preQ1 were found in the room-temperature MD simulations. These results are in good agreement with the structural analysis that preQ1 forms important interactions with the riboswitch. The experimental data also indicate that the triple stacks of U9-A51-U18, C8-preQ1-U19, and U10-A52-U17 can stabilize the binding pocket, which is consistent with our simulations that preQ1 can form stable hydrophobic interactions with U19, A51, C20, and U9 (supplementary Figure S6). The previous experiment also suggests that A35 and A50 act as screw cap and block the ligand from escaping the binding pocket. The networks and communities of A35G and A50G mutants suggest that the information flow fails to transfer within the riboswitch. This indicates that A35 and A50 are hub nodes and are critical for information transfer. This is consistent with the experimental observation^15^.

## Conclusion

Nucleotide/nucleotide fluctuation correlation network was used to research the allosteric switch mechanism of preQ1 riboswitch. The results suggest that the correlation network of the bound riboswitch has more hub nodes than that of the apo state. The community network of the bound state is clustered into an intact community. However, the P4 helix is isolated from the community upon dissociation from preQ_1_. The information flow can freely transfer from the binding pocket of preQ1 to the expression platform of the P3 helix. On the contrary, information flow can hardly transfer from the P4 helix to the expression platform in the apo state. Therefore, a hypothesis of “preQ1-binding induced allosteric switching” is used to explain the preQ1 binding and switch regulation. These observations were further confirmed with the results from community analysis based on the Girvan-Newman algorithm for multiple mutants and network-weakened systems. Finally, a possible allosteric pathway including A50 and A51 is also identified based on the analysis of the network for the bound state and confirmed by mutations and network perturbation. Interestingly, no pathways could be found in the apo state or any disruptive mutants.

## Additional Information

**How to cite this article**: Wang, W. *et al*. Dynamics Correlation Network for Allosteric Switching of PreQ_1_ Riboswitch. *Sci. Rep.* **6**, 31005; doi: 10.1038/srep31005 (2016).

## Supplementary Material

Supplementary Information

## Figures and Tables

**Figure 1:**
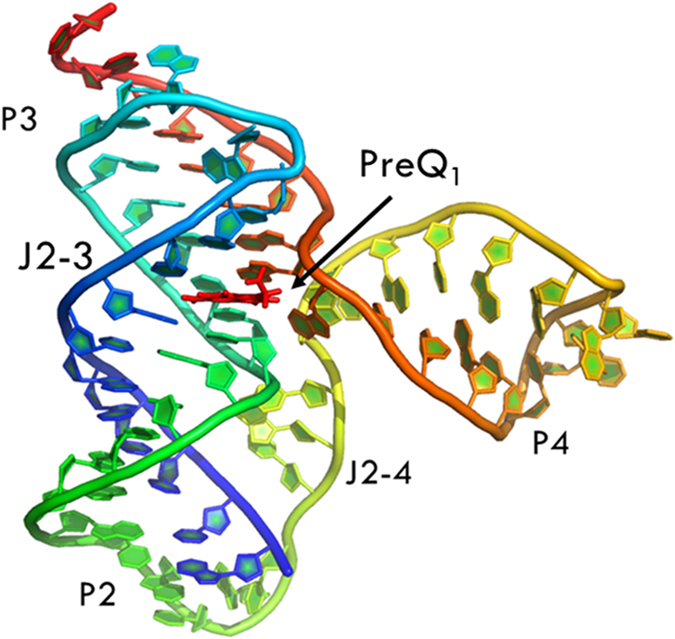
Ribbon representation of the NMR structure for the preQ1 riboswitch. Helices P2, P3, and P4 (light green, cyan and light red), pseudoknots J2-3 and J2-4 (blue and yellow), preQ1 (red) are labeled.

**Table 1:** Simulation conditions for all systems.

System	Mutant	Temp. (K)	Time (ns)	Traj. Num.	Ions	Waters
Apo riboswitch	WT	298	100	3	58 Na+	8738
Bound riboswitch	WT		100	3	57 Na+	8746
M1	G5C/U6A		100	1	57 Na+	9541
M2	G5C/U6A/A21U/C22G		100	1	57 Na+	8678
M3	A50U/A51U		100	1	57 Na+	9608
M4	A50U/A51U/U18A/U19A		100	1	57 Na+	8607
M5	G39C/G40C		100	2	57 Na+	8998
M6	G39C/G40C/C45G/C46G		100	2	57 Na+	8645
MS1	A35G		100	2	57 Na+	9836
MS2	A50G		100	2	57 Na+	8652

**Table 2:** Topology parameters of wild type and mutant networks.

System	Clustering coefficient	Network density	Network centralization	Network heterogeneity	Avg. number of neighbors
Apo riboswitch	0.405	0.060	0.045	0.325	3.458
Bound riboswitch	0.479	0.069	0.068	0.358	4.10
M1	0.477	0.064	0.076	0.334	3.729
M2	0.478	0.072	0.066	0.312	4.233
M3	0.422	0.067	0.053	0.325	3.967
M4	0.468	0.063	0.022	0.244	3.733
M5	0.437	0.059	0.044	0.347	3.467
M6	0.441	0.066	0.039	0.312	3.831
MS1	0.449	0.065	0.044	0.382	3.649
MS2	0.449	0.069	0.086	0.369	4.10
