# Supplementary material for: Dynamics Correlation Network for Allosteric Switching of PreQ1 Riboswitch
Source: Sci Rep. 2016 Aug 3;6:31005. doi: 10.1038/srep31005 (PMC4971525; doi:10.1038/srep31005)
Supplement: Supplementary Information [file srep31005-s1.pdf]

Supplementary Materials

Dynamics Correlation Network for Allosteric Switching of PreQ$_1$ Riboswitch

Wei Wang,[1,#] Cheng Jiang,[1,#] Jinmai Zhang,[1] Wei Ye,[1]
Ray Luo,[2,*] and Hai-Feng Chen[1,3,*]

[1]State Key Laboratory of Microbial metabolism, Department of Bioinformatics and Biostatistics, College of Life Sciences and Biotechnology, Shanghai Jiaotong University, 800 Dongchuan Road, Shanghai, 200240, China

[2]Departments of Molecular Biology and Biochemistry, Chemical Engineering and Materials Science, Biomedical Engineering, University of California, Irvine, California 92697-3900, USA

[3]Shanghai Center for Bioinformation Technology, 1278 Keyuan Road, Shanghai, 200235, China

[#] These authors contributed equally to this work.

*****Corresponding authors
Email addresses: haifengchen@sjtu.edu.cn; rluo@uci.edu
Tel: 86-21-34204348
Fax: 86-21-34204348.

The authors declare that there is no conflict of interest.

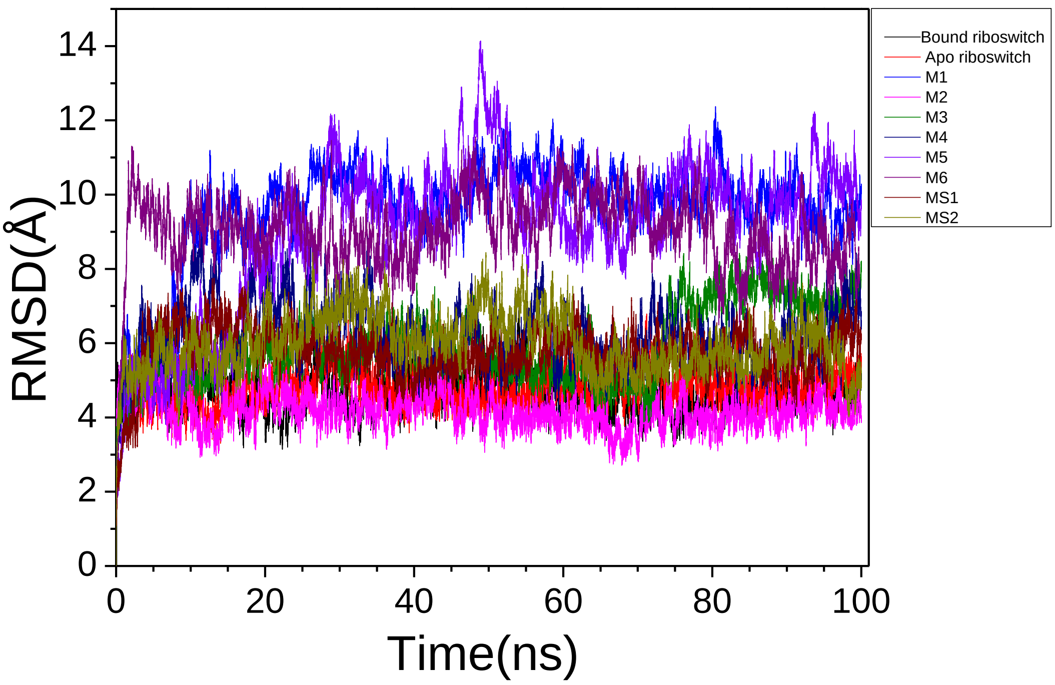

Figure S1. C5’ RMSD for all simulation systems.

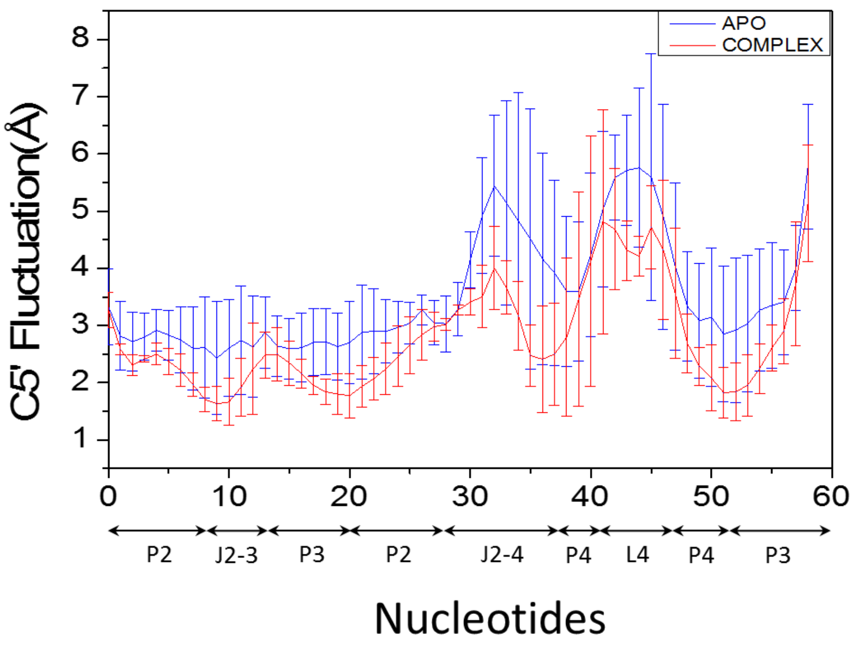

Figure S2. C5' fluctuation for apo and bound riboswitches.

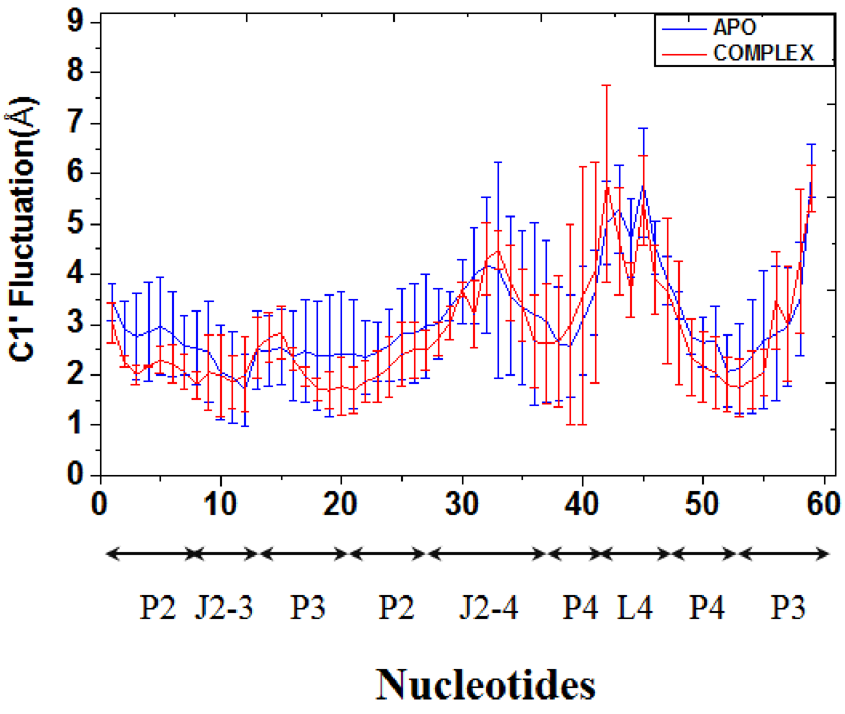

Figure S3. C1' fluctuation for apo and bound riboswitches.

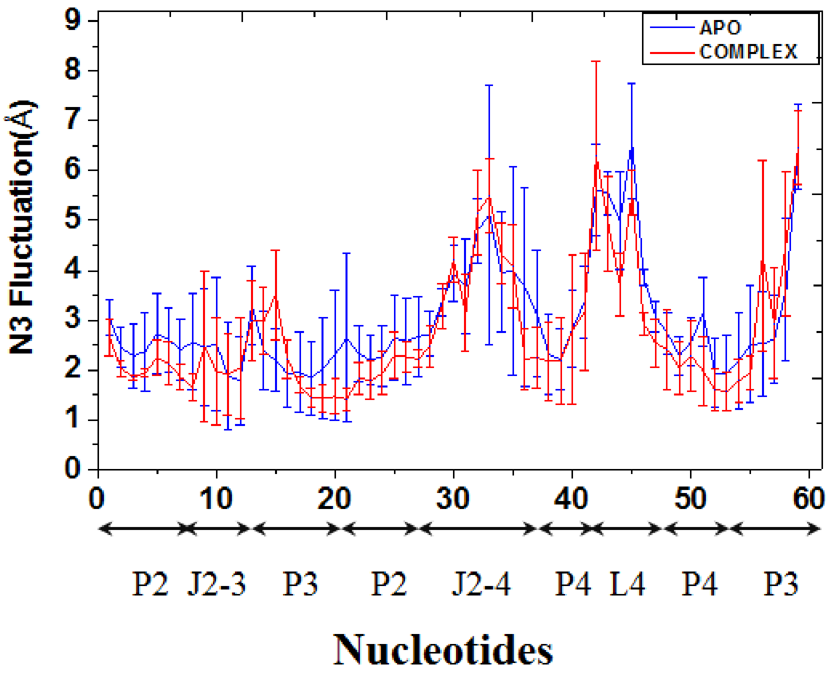

Figure S4. N3 fluctuation for apo and bound riboswitches.

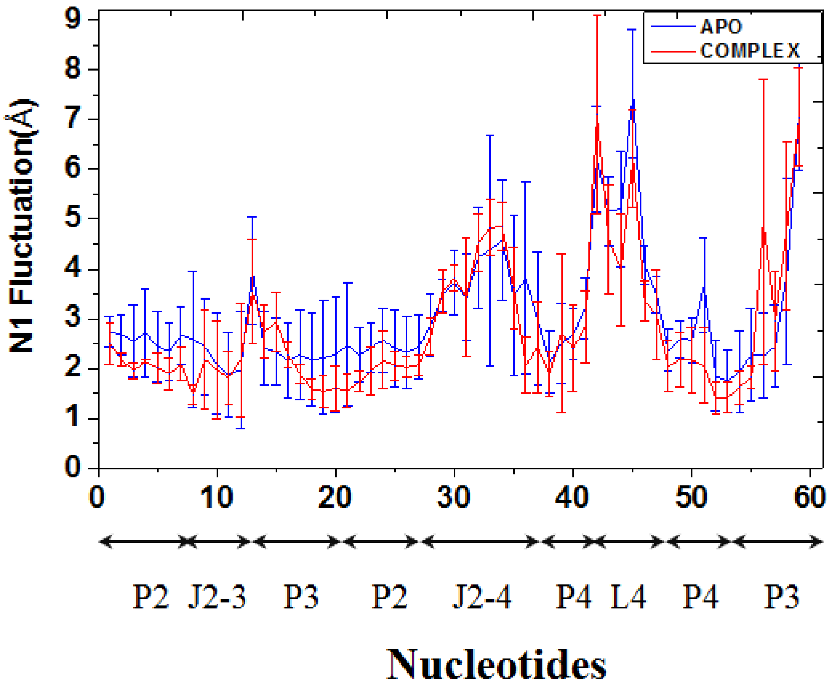

Figure S5. N1 fluctuation for apo and bound riboswitches.

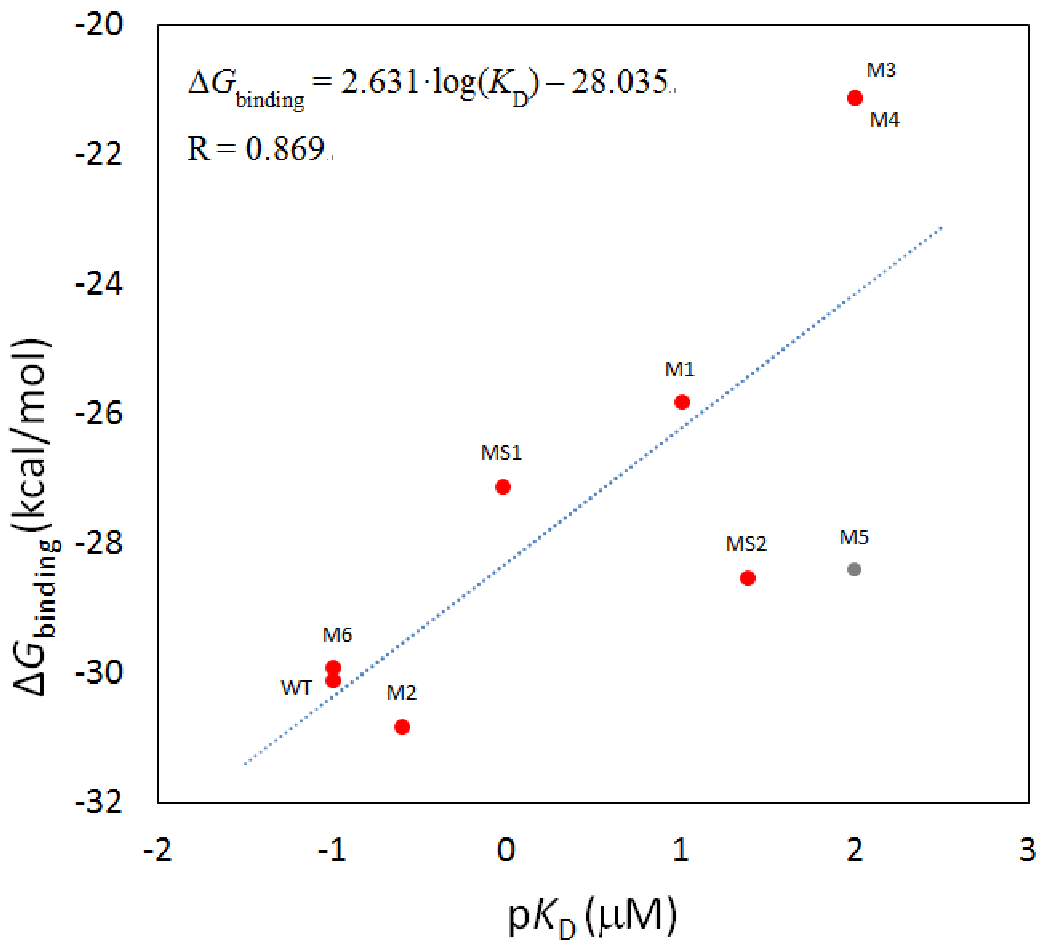

Figure S6. Correlation between measured dissociation constants and MMPBSA binding free energies. M5 is the outlier. The correlation coefficient R appears not too strong correlation, however the limited confidence can be found between binding free energy and K_D.

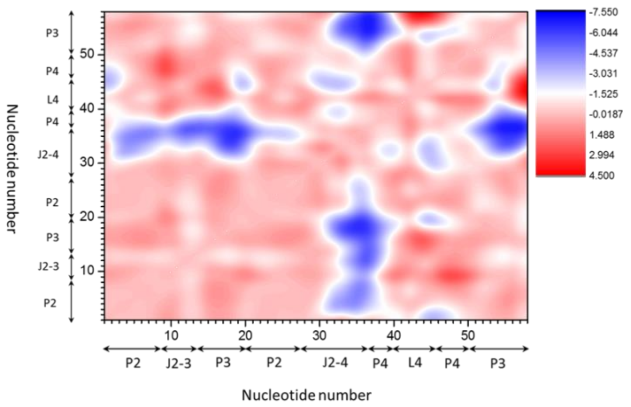

Figure S7. Distance different landscape between apo and bound riboswitches.

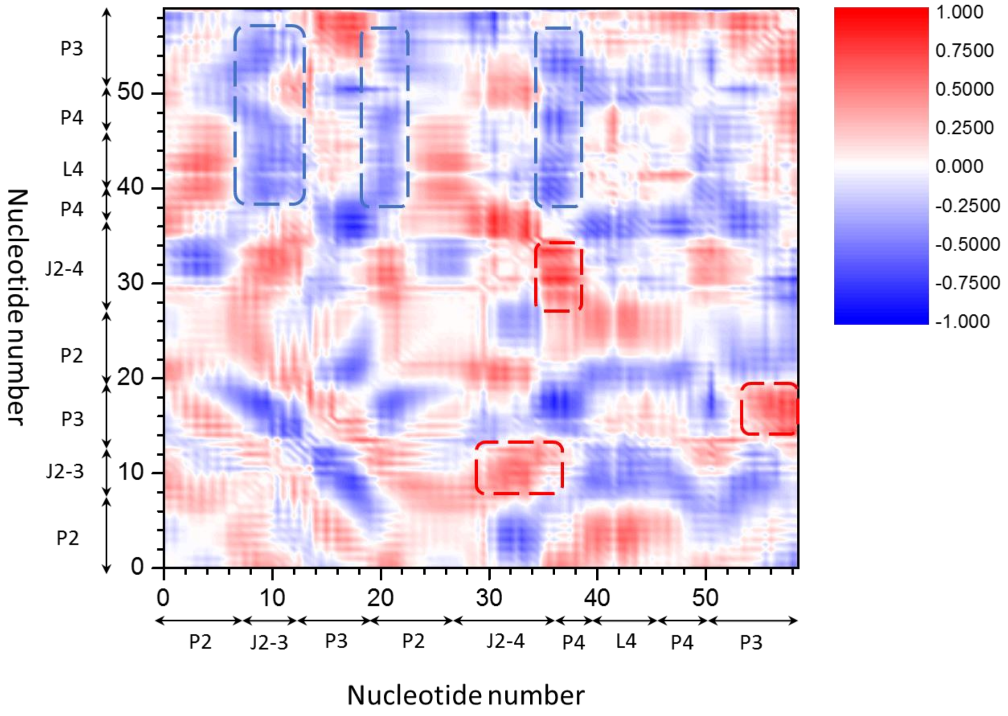

Figure S8. Correlation differences between apo and bound riboswitches (C_{apo} minus C_{bound}), secondary structures were labelled.

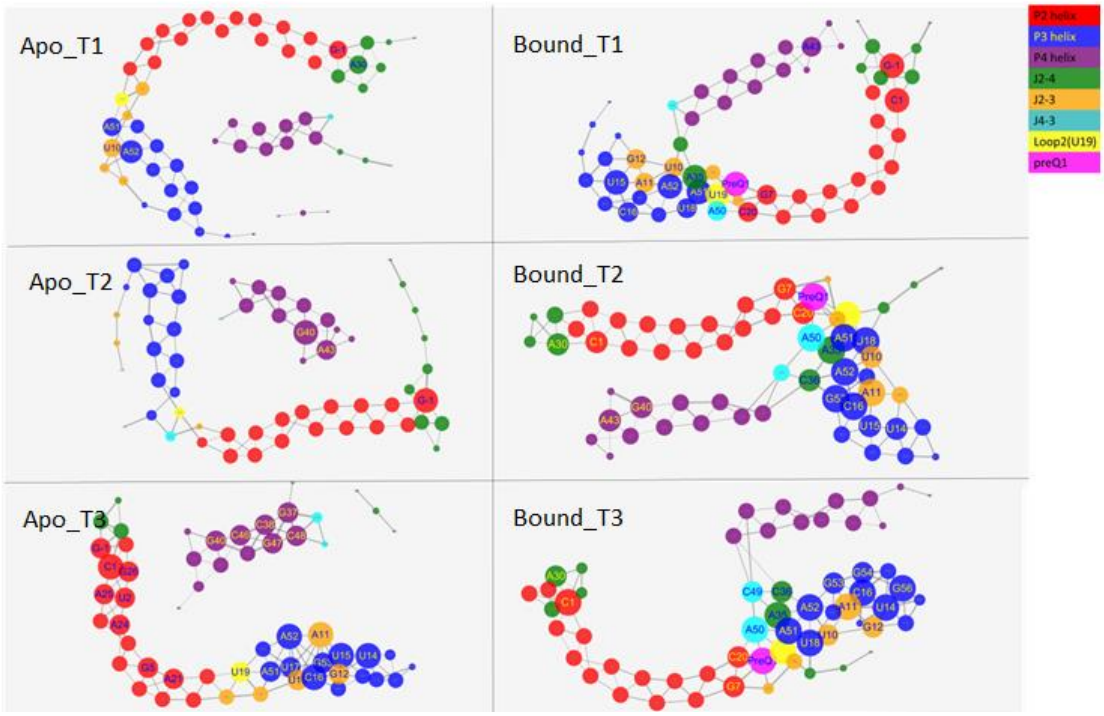

Figure S9. Dynamics correlation networks for three independent trajectories of apo and bound riboswitches.

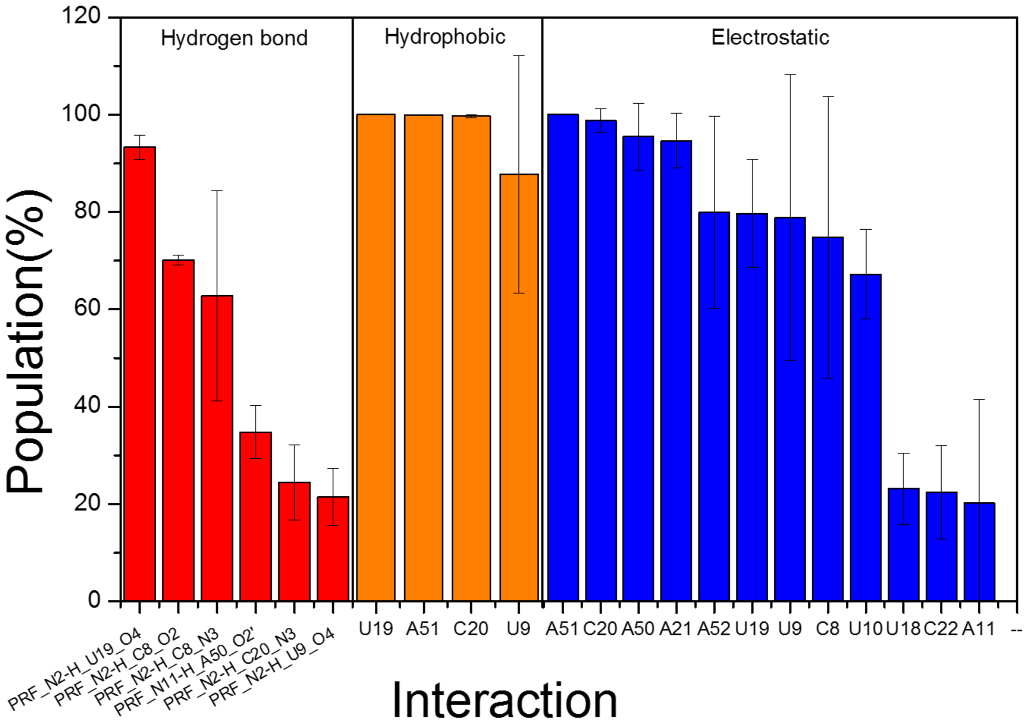

Figure S10. Interaction between preQ1 and riboswitch for wild type.

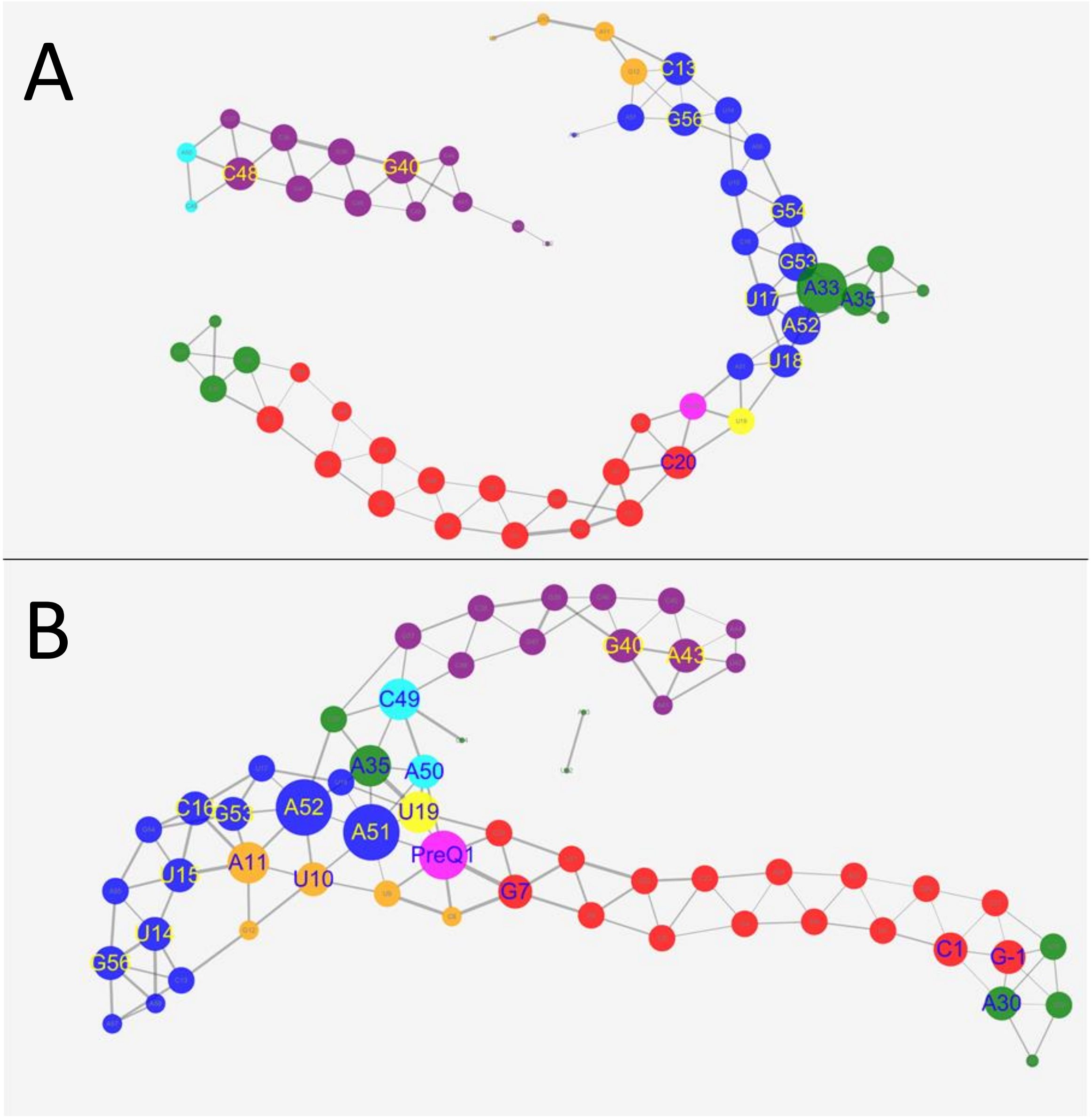

Figure S11. Correlation networks of M1 and M2 mutants. A: M1 mutant. B: M2 mutant.

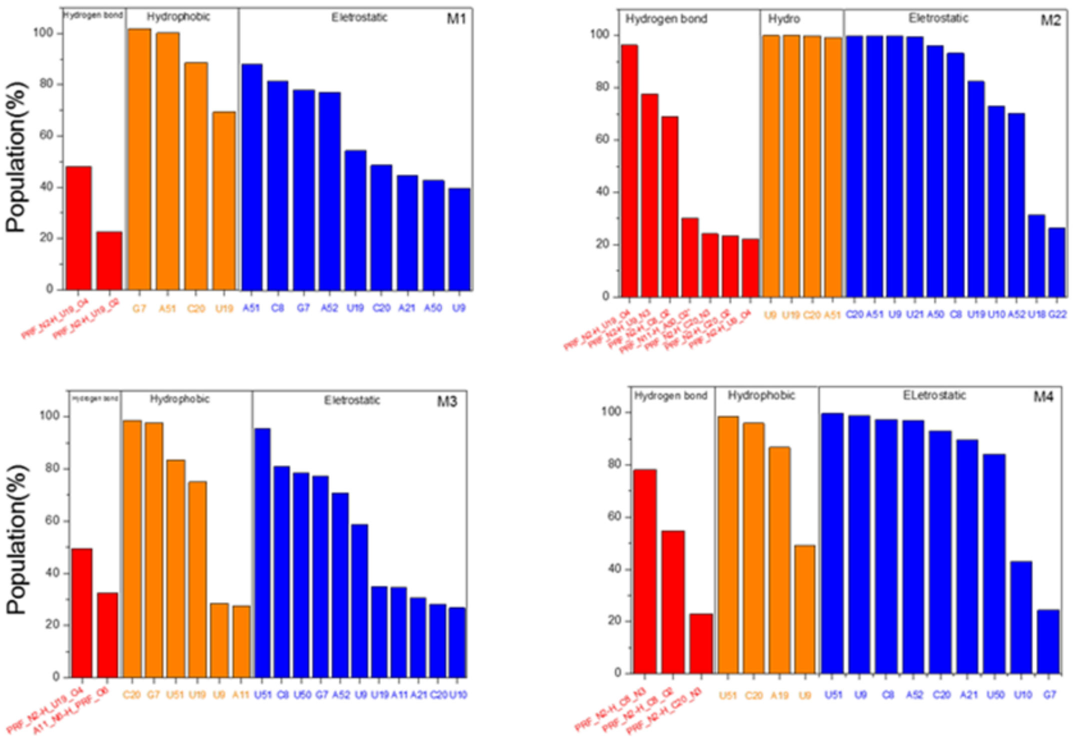

Figure S12. Interactions between preQ1 and riboswitch for mutants.

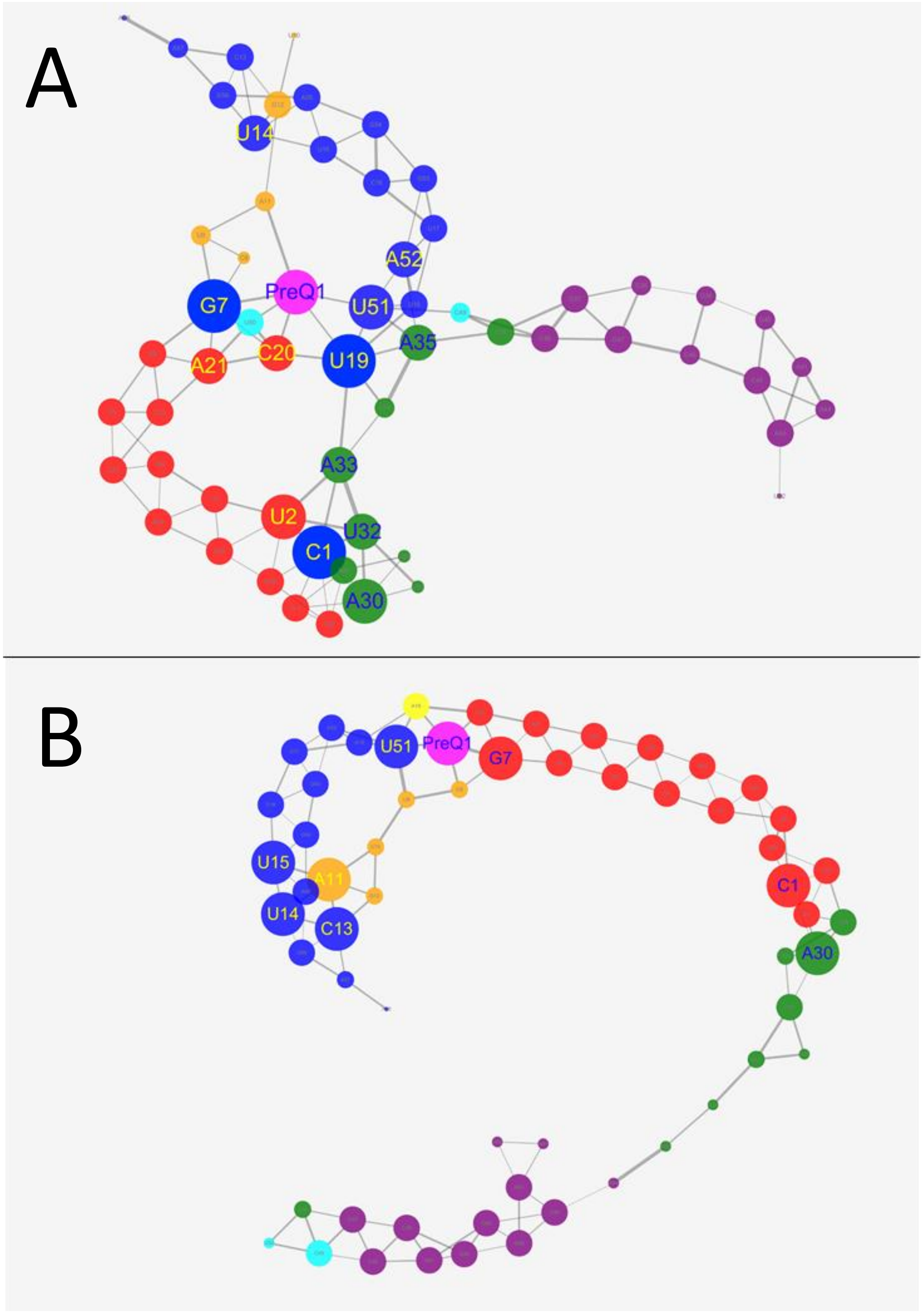

Figure S13. Correlation networks of M3 and M4 mutants. A: M3 mutant. B: M4 mutant.

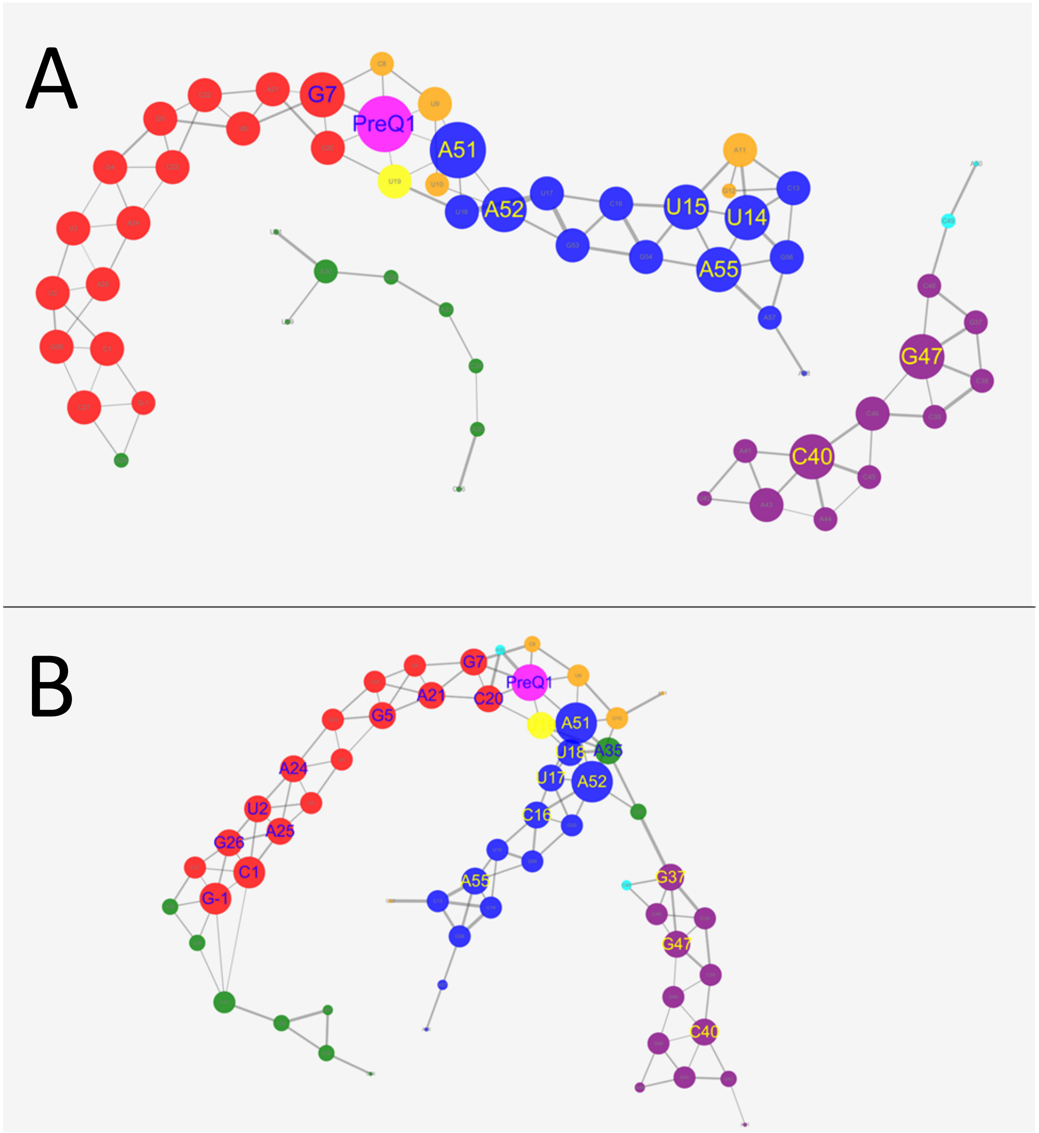

Figure S14. Networks of M5 and M6 mutants. A: M5 mutant. B: M6 mutant.

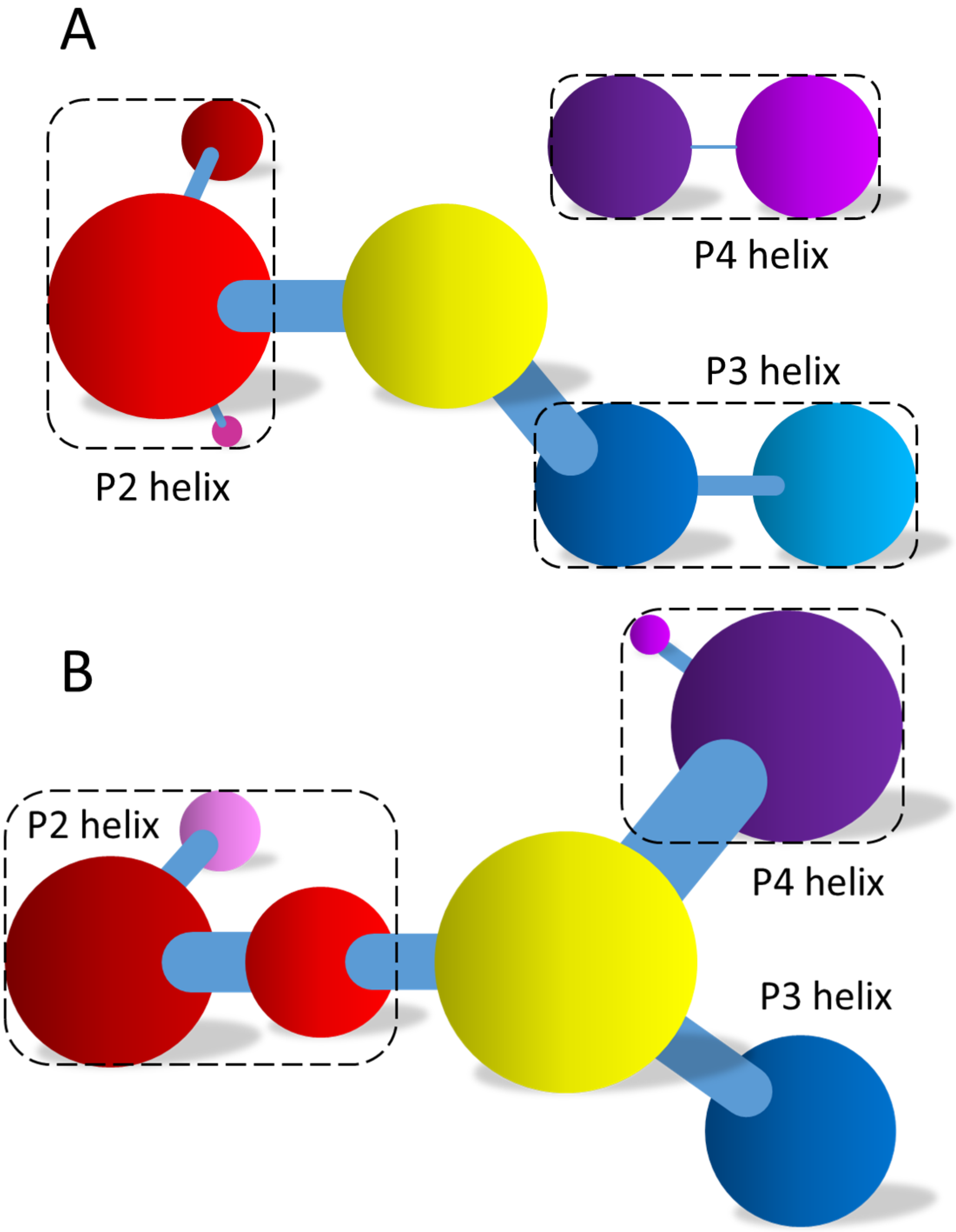

Figure S15. Community networks of M5 and M6 mutants. A: M5 mutant. B: M6 mutant.

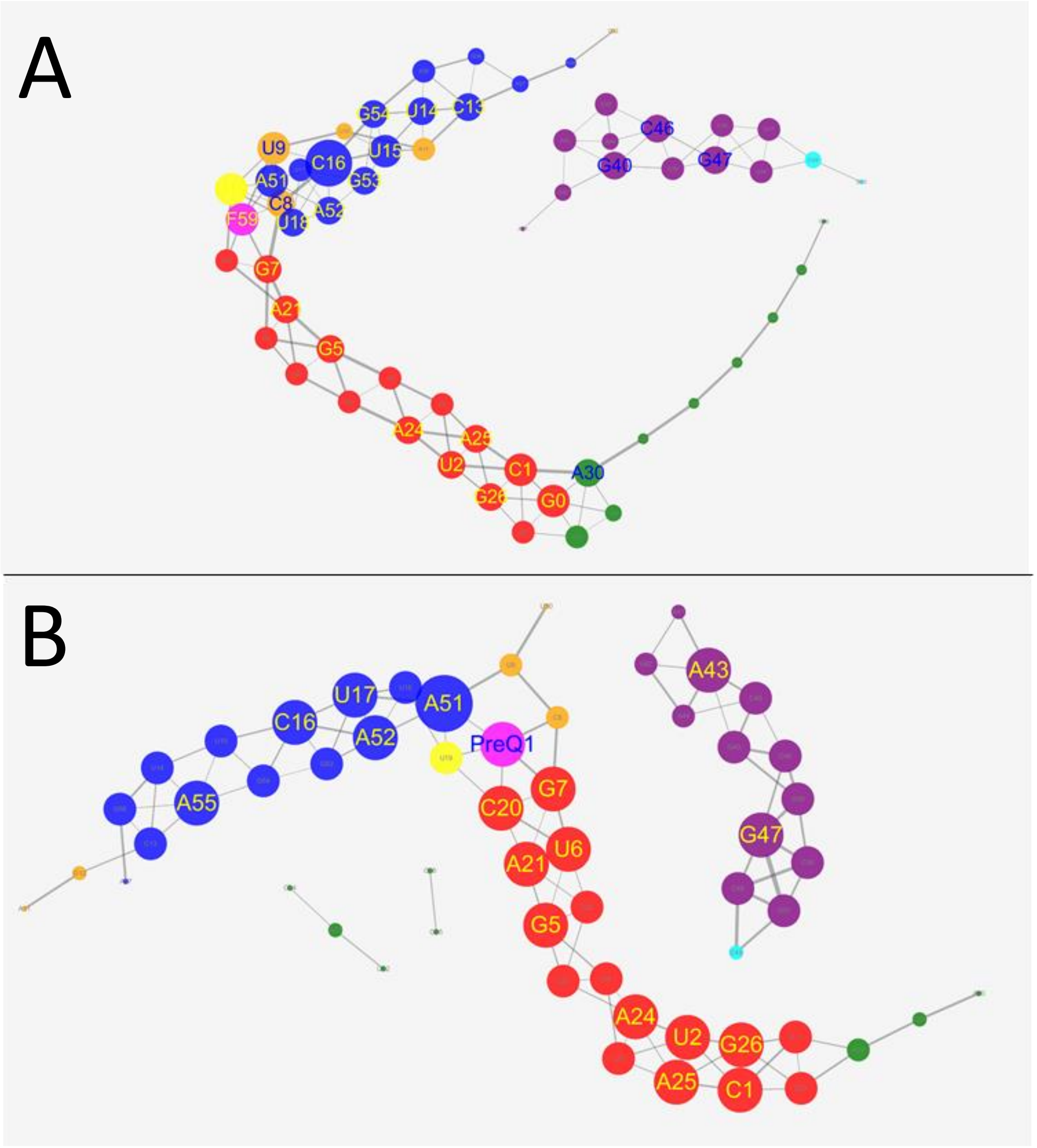

Figure S16. Correlation network of A50G and A35G mutants. A: A50G mutant. B: A35G mutant.

Table S1. Binding free energy of all systems.

System	Mutant	Binding free energy (kcal/mol)	K_D
WT	/	-30.1±3.8	100nM
M1	G5C/U6A	-25.8±3.4	10 μM
M2	G5C/U6A/A21U/C22G	-30.8±2.8	250nM
M3	A50U/A51U	-21.1±4.6	>100 μM
M4	A50U/A51U/U18A/U19A	-21.1±3.7	>100 μM
M5	G39C/G40C	-28.4±3.0	>100 μM
M6	G39C/G40C/C45G/C46G	-29.9±2.9	~100nM
MS1	A35G	-27.1±3.3	950nM
MS2	A50G	-28.5±3.0	24 μM

Table S2. *P*-values of *KS* test for three independent trajectories of apo and bound riboswitches.

		Apo			Bound		
	Trajectory	T1	T2	T3	T1	T2	T3
Apo	T1	-	-	-			
	T2	0.50	-				
	T3	0.72	0.11	-			
Bound	T1	0.033	0.032	0.035	-		
	T2	0.003	0.010	0.035	0.12	-	
	T3	0.005	0.010	0.035	0.38	0.99	-
